# Comparing the effectiveness of single-lumen high-frequency positive pressure ventilation with double-lumen endobronchial tube for the anesthesia management of endoscopic thoracic sympathetic blockade surgery

**DOI:** 10.1097/MD.0000000000035315

**Published:** 2023-10-13

**Authors:** İlhan Akaslan, Suna Koc

**Affiliations:** a Department of Thoracic Surgery, Biruni University, Istanbul, Turkey; b Department of Anesthesiology and Reanimation, Biruni University, Istanbul, Turkey.

**Keywords:** double lumen intubation, endoscopic thoracic sympathetic blockage, facial flashing, high-frequency positive pressure ventilation, hyperhidrosis, single lumen intubation ventilation

## Abstract

**Objectives:**

In this trial, we aimed to compare anesthetic effectiveness of single lumen tube (SLT) for tracheal intubation with high-frequency positive pressure ventilation (HFPPV) versus classic double lumen tube (DLT) for tracheal intubation in endoscopic thoracic sympathetic blockade surgery.

**Design:**

This was a prospective randomized controlled clinical study.

**Setting:**

The study was single-centered and conducted in a university hospital.

**Participants:**

There were 135 endoscopic thoracic sympathetic blockade patients in this study.

**Interventions:**

The patients were randomly allocated either to DLT (n = 67) or SLT (n = 68) groups. In SLT group, the ventilator setting was kept with frequencies that range from 1 to 1.8 Hz (60–110/min). Data regarding anesthesia duration, surgery duration, difficult intraoperative lung deflation, postoperative atelectasis, postoperative pain, postoperative pneumothorax were recorded and compared. All patients were operated by a single experienced surgeon under general anesthesia provided by the same anesthesia team.

**Measurements and main results:**

Both groups were age and gender matched. Among all recorded variables, only anesthesia time was found to be close to statistical significance (*P* = .059, favoring single lumen). All other parameters were found to be similar between groups. (*P* < .05).

**Conclusion:**

We reported that DLT and single lumen tracheal intubation were equally effective for lung deflation during surgery, and SLT with HFPPV ventilation mode during endoscopic thoracic sympathetic blockade surgery provided the surgeon with an adequate and clean workspace with shorter onset of anesthesia. We may suggest the HFPPV technique for uncomplicated surgery groups or where sufficient conditions for DLT cannot be provided in the operating room.

**Highlights**

- Single lumen intubation HFPPV provides the same level of surgical comfort as double lumen.
- The rate of postoperative pneumothorax and atelectasis is similar in DLT and SLT.
- Double lumen requires slightly longer anesthesia time, but this finding is probably related to the anesthesiologist’s experience.

## 1. Introduction

Endoscopic thoracic sympathetic blockage (ETSB) is a commonly performed procedure for treating conditions like palmar hyperhidrosis (PH), facial flashing (FF), and hyperhidrosis, Raynaud’s syndrome, social phobia, and arrhythmia treatment.^[1–3]^ Although several reports have published the feasibility of local anesthesia for this type of surgery, it is usually performed under general anesthesia with endotracheal intubation. The procedure is performed through an artificially made pneumothorax in the pleural cavity and this space allows visualization of the target area as well as surgical manipulation. The larger space provides better surgical conditions. However, lung deflation may cause desaturation during surgery which may increase the rate of postoperative atelectasis.^[3]^

There are 2 main tracheal intubation methods in thoracic surgery—double lumen tube (DLT) and single lumen tube (SLT). DLT is a standard choice for open and endoscopic thoracic surgery. It allows separate ventilation of both lungs as well as rapid and effective deflation/inflation of the lung as necessary. The main advantage of DLT is the ability to provide an ample space for the surgeon to visualize the target area and perform the procedure in a comfortable manner. However, there are authors who have advocated the use of SLT for minor, short-duration video assisted thoracic surgeries like ETSB.^[4]^ Although SLT does not provide as rapid and effective deflation as DLT, the pneumothorax cavity is usually enough to perform the procedure.^[4]^ The advantages of SLT include shorter anesthesia duration, no need for sophisticated medical equipment like fiber optic bronchoscope and experience of anesthesiologist, lower cost, and less complication rate. Nonetheless, these benefits should not compromise the safety and effectiveness of the procedure.^[4]^

Also new approaches like lung ultrasound develop to evaluate the performance o for DLT position in one lung ventilation. A recent meta-analysis reported better specificity with similar sensitivity for lung ultrasound when compared with clinical evaluation. And authors suggested ultrasound should be considered as a suitable imaging tool.^[5]^

High-frequency positive pressure ventilation (HFPPV) was first defined in 1967 to minimize the effect ventilator associated variables like cardiac output and mean arterial pressure and by lowering peak airway pressures.^[6–8]^ These pressures were accomplished with low tidal volumes and high breathing frequency rate for ventilation involving o high-pressure gas insufflation. They suggested an inspiratory gas with frequency of 55 to 110 times/min.^[7,8]^

In our experience, the main factor impeding surgery is difficult lung deflation (DLD). In theory, DLT is the superior technique for surgery, but we planned to demonstrate that if HFPPV mode with SLT may provide similar benefits for uncomplicated cases. We aimed to compare anesthetic effectiveness of SLT for tracheal intubation with HFPPV versus classic DLT for tracheal intubation in ETSB surgery. Our secondary aim was to compare complications of the techniques.

## 2. Materials and methods

This was a prospective randomized study approved by Biruni University Institutional Review Board (no: 2019/27-27). All patients provided were given informed consent regarding the surgical procedure and enrollment into the study. 135 patients underwent bilateral ETSB procedure under general anesthesia. The patients were randomly assigned to DLT (n = 67) and SLT (n = 68) groups.

### 2.1. Preoperative assessment

All patients had failed conservative medical treatment for underlying conditions prior to surgery. A thorough clinical examination was performed on all patients, which included evaluation of the sympathetic activity. The clinical evaluation involved assessment by 3 additional specialists: dermatologist, neurologist, and psychiatrist to rule out non-surgical candidates.

### 2.2. The procedure

All patients were managed by the same anesthesia team. The patients in the DLT group were intubated by using Sher-I-Bronch® left-sided endobronchial tube (Sheridan Catheter Corp, Argyle, NY). The patients in the SLT group were intubated by using standard endotracheal tube.

#### 2.2.1. Anesthesia management.

Anesthesia induction and maintenance of both groups were performed in the same way. General anesthesia was induced with propofol (1–2 mg/kg) + fentanyl (1 µg/kg) and rocuronium (0.5–1.0 mg/kg). Patients were intubated in supine positions and then placed in a semi-fowler position with arms abducted. Anesthesia maintenance was provided with sevoflurane (1 MAC) and 50% O_2_/air mixture.

Patients in the DLT group were blocked on the right side in the standard right procedure and the left side in the left procedure. (With DLTs with male number 39 and female number 37). Accuracy of tube placement was controlled by osculation and fiberoptic analysis if in doubt.

Patients in the SLT group were intubated with a normal, single lumen endotracheal tube (number 8.0 for male and number 7.5 for female). The tube position was checked after each repositioning of the patients.

In the beginning, mechanical ventilation strategies of the patients were the same in both groups, they were as follows. A frequency of 12 to 15 per minute was adjusted to achieve an end-tidal CO_2_ of 35 to 45 mm Hg with a tidal volume of 4 to 6 mL/kg and a plateau pressure lower than 35 cm H_2_O until thoracoscopy ports were placed. While the thoracoscopy ports were placed, the ventilator was separated from the patient and the lungs were deflated, allowing the ports to be settled safely.

In the SLT group, after thoracoscopic input, the anesthetist was in contact with the surgeon and his lung pressures were reduced until the lowest pressure, where the surgeon’s vision was optimal. The ventilator setting was kept at this pressure, by switching to HFPPV mode under constant fresh flow HFPPV at frequencies that range from 1–1.8 Hz 60–110/min.^[8]^

In the DLT group, the lung on the side to be treated was distinguished from the ventilator and deflated and single lung ventilation was performed with the other lung. Tidal volume reduced to 1 to 2 mL/kg, positive end expiratory pressure 8 cm H_2_O pressure, frequency increased to 14 to 16 per minute and fraction of inspired oxygen increased from 50% to 70%. SpO_2_ is balanced so that it does not fall below 90%.

At the end of the surgical procedure, the lungs were observed under thoracoscopy and in the DLT group, double lung was switched, and lung pressures and tidal volumes were manually increased in both groups until there was no atelectasis lung area. After the procedure, all patients were put in supine position and antagonized with standard neostigmine 0.05 mg/kg + atropine 0.01 mg/kg and were dismissed.

### 2.3. Surgical procedure

All patients were operated on by the same surgeon. The patients were placed into a semi-fowler position with arms abducted. Two thoracoscopic ports were inserted: the first – at the intersection of the anterior axillary line with pectoralis major muscle border; the second—on the anterior axillary line at 5th of 6th intercostal space. The first port was used for surgical instruments and the second for the thoracoscope. Following the entry into the pleural cavity the lung usually deflated providing ample space for manipulation. However, in some cases the lung didn’t deflate immediately. In these cases, gentle pressure was applied to the lung to facilitate its collapse. The cases which took *more than 5 minutes* to achieve sufficient deflation were designated as DLD. The sympathetic chain was carefully exposed by opening the parietal pleura and clipped by medium hemostatic titanium clips (Peters Surgical, Bobigny Cedex, France). A catheter was inserted into the thoracic cavity and kept at negative pressure. The lung was inflated, and residual air was drained via the catheter. The catheter was removed, and the surgical wound was closed.

All patients were followed in a surgical ward after the procedure. Postoperative postero-anterior chest films were obtained, and all patients were discharged on the same day.

### 2.4. Data acquisition

The data were collected and used for statistical analysis included: age, gender, total anesthesia duration, surgical time, operating theater occupancy time, difficult lung deflation incidence, postoperative atelectasis incidence, postoperative pneumothorax incidence, postoperative pain visual analog scale, and total hospital admission time. Total anesthesia duration included the sum of induction (time between the onset of anesthesia and the correctness of the intubation tube) plus wake-up times. All time variables were collected by either anesthesia or surgical duration and recorded independently.

Postoperative atelectasis and pneumothorax were evaluated by the single radiologist. on anterior-posterior chest X-ray. Postoperative pain scoring was performed by surgical ward nurses using standard visual analog scale score. Hospital admission durations were obtained from patients’ electronic medical records.

We evaluated surgeon satisfaction with 5-point Likert scale (Excellent-Good-Fair-Poor-Very Poor).

#### 2.4.1. Sample size calculation.

We made the sample size analysis with the Power and Sample Size Program (PS version 3.1.2). We utilized the study of Toolabi et al^[9]^ for thoracoscopic sympathectomy surgery time data to calculate our power analysis and a 10% difference was predicted for the evaluation. Which showed that the difference in the response of pairs was normally distributed with SD of 11. If the true difference of mean response of matched pairs was 4, we assessed 122 subjects to be able to reject the null hypothesis with power of 0.8. The Type I error probability associated with this test of this null hypothesis was 0.05. When 10% dropouts were estimated at least 134 patients were planned to include.

### 2.5. Statistical analysis

The data was analyzed using Statistical Analysis in Social Science software (version 24.0; IBM, Armonk, NY). Descriptive methods were used for assessing statistical data of the study and stated in terms of mean, standard deviation, and frequency. The paired *t* test and chi-square test were used for the normal distribution of the parametric data and the Mann–Whitney *U* test was used when for the evaluation of non-parametric data. *P* value less than < .05 was considered statistically significant.

## 3. Results

In this study, a total of 135 patients were included to the analyses. The reason for surgery was PH in 75, facial flushing in 55 and both (PH + FF) in 5 patients. (Table 1).

**Table 1:** The distribution of surgical indications among treatment groups.

	Palmar hyperhidrosis	Facial flashing	PH + FF	Total
DLT	37	28	2	67
SLT	38	27	3	68
Total	75	55	5	135

DLT = double lumen tube, FF = facial flashing, PP = palmar hyperhidrosis, SLT = single lumen tube.

We reported the mean age of all patient groups as 26.19 ± 7.16 years. From the participants 82 (60%) were male and 53 (40%) were female. All demographic data can be found in Table 2.

**Table 2:** Demographic data of all patient groups.

Data	Number (%)
Age (yr)	26.19 ± 7.16
Group	
SLT	68 (50.4)
DLT	67 (49.6)
Gender	
Female	54 (40.0)
Male	81 (60.0)
VAS score	4.30 ± 1.75
Surgeon satisfaction	4.76 ± 0.53
Operation time	41.04 ± 11.21
Anesthesia time	30.17 ± 11.39
Atelectasis	
Yes	19 (14.1)
No	116 (85.9)
Difficult lung deflation	
Yes	15 (11.1)
No	120 (88.9)

DLT = double lumen tube, SLT = single lumen tube, VAS = visual analog scale.

The distribution of cases was similar in both groups. Anesthesia time which included induction and wake-up times were slightly longer in the DLT group (32.04 ± 12.99 vs. 28.33 ± 9.30) and the difference was near significant levels (*P* = .059). Surgical time was found to be minimally elevated in the SLT group (40.37 ± 10.49 vs 41.70 ± 11.93) and the difference was not statistically significant (*P* = .492). All other parameters also demonstrated non-significant differences between treatment groups (Table 3).

**Table 3:** The comparison of data in terms of groups.

	DLT (n = 67)	SLT (n = 68)	*P* value	95% confidence interval of the difference	
				Upper	Lower
Age (yr)	25.65 ± 6.28	26.72 ± 7.94	.390	1.370	3.509
Gender					
Male	39 (58.20%)	42 (62.68%)	.674	−0.128	0.198
Female	28 (41.80%)	26 (37.32%)			
Anesthesia time (min)	32.04 ± 12.99	28.33 ± 9.30	.059	−7.54959	0.13651
Surgical time (min)	40.37 ± 10.49	41.70 ± 11.93	.492	−2.49436	5.15986
Difficult lung deflation	7 (10.44%)	8 (11.76%)	.808	−0.096	0.122
Atelectasis	3 (4.47%)	6 (8.82%)	.311	−0.164	0.074
Pneumothorax	1 (1.49%)	0	.312	−0.034	0.0063
Postoperative pain (VAS)	4.41 ± 1.96	4.19 ± 1.51	.283	−0.82367	0.37020
Total length of hospital stay (h)	16.51 ± 6.36	17.58 ± 6.44	.335	−1.11539	3.24818
Surgeon satisfaction	4.73 ± 0.56	4.79 ± 0.50	.498	−0.11989	0.245446

DLT = double lumen tube, SLT = single lumen tube, VAS = visual analog scale.

*P < .05 accepted as significant.

In terms of complications, we did not report any significant differences (*P* > .05). Difficult lung deflation, atelectasis and pneumothorax rates were similar among groups. Also we did not report any surgical complications.

## 4. Discussion

Double lumen intubation was a standard choice for ETSB surgery during the early years.^[10,11]^ However, the use of SLT is not novel, since the first reports date back to the early 1990’s.^[11,12]^ The following studies also reported satisfactory results with SLT during ETSB surgery.^[4,12–16]^ Nevertheless, most of these articles report retrospective single arm studies and very few prospective studies were conducted on this matter. Two early studies comparing DLT and SLT demonstrated contradictory results in terms of intraoperative oxygenation,^[17,18]^ adding confusion to this topic. Subsequently, Wong et al^[14]^ studied the effect of DLT and SLT on intraoperative cardio-respiratory parameters in a small retrospective study. The authors found that oxygenation and cardiovascular stability were better in the SLT group. Likewise, in a similarly sized small, prospective, non-randomized trial, Toolabi et al^[9]^ demonstrated the superiority of SLT over DLT. Due to lack of evidence hitherto, even most recent articles report DLT on routine basis.^[19,20]^ To our knowledge, the present study is the largest, prospective, randomized trial on this matter.

In our patients with SLT and lung deflation problems, we employed HFPPV and provided an adequate working area for the surgeon in short-term video assisted thoracic surgeries operations. With this technique, we did not observe a difference in oxygenation between the patients in whom we applied SLT and those in whom DLT was applied.

Similar to previous reports, we found that the choice of intubation affects neither surgical comfort nor the postoperative complication rate. DLD is the most challenging obstacle and therefore was the primary focus of this trial. Our results indicate that the choice of intubation does not affect the incidence of DLD. This finding indicates that DLD is probably due to the patient’s unrecognized constitutional factor(s). Further research on this matter is required to shed light on the underlying mechanism of intraoperative lung resistance.

Several studies compared postoperative anesthesiologic complications of DLT versus SLT. For example, it is well known that the incidence of airway damage is higher in DLT patients.^[21]^ Thus, to avoid redundancy, we did not include upper airway assessment in our study. However, there are few studies investigated postoperative atelectasis. Atelectasis is a frequent complication of lung deflation, and it has been speculated that its incidence correlates with the magnitude and duration of intraoperative lung deflation. As DLT provides better deflation, one would expect that the incidence of postoperative atelectasis would be higher. Interestingly, we found the SLT group had twice as much atelectasis rate as DLT, yet the difference was not statistically significant.

DLT is considered as more time and effort consuming than SLT. To test this hypothesis, we recorded total anesthesia time and found minimal difference favoring SLT. Although the difference wasn’t statistically significant (*P* = .059) the data indicate that DLT is indeed more time-consuming. The difference is expected to be small with anesthesiologist’s experience. Since our institution has a high volume of routine DLT’s, it is not surprising, why anesthesia durations were close between groups.

This study has several limitations. First, it was performed at a single institution. Therefore, the multi-center study is required to rule out institution-related bias. Second, for obvious reasons neither anesthesiologist nor the surgeon were blinded to treatment groups which adds bias to our study. And also, the number of patients in our study is another serious limitation. Another limitation to our study was we did not no compare between intraoperative oxygen saturation and blood gas carbon dioxide partial pressure. Because high-frequency ventilation may affect ventilation function, leading to hypoxia and carbon dioxide accumulation.

In conclusion, We reported that DLT and single lumen tracheal intubation were equally effective for lung deflation during surgery, and SLT with HFPPV ventilation mode during ETSB surgery provided the surgeon with an adequate and clean workspace with shorter onset of anesthesia. We may suggest the HFPPV technique for uncomplicated surgery groups or where sufficient conditions for DLT cannot be provided in the operating room.

## Author contributions

**Conceptualization:** Suna Koc.

**Data curation:** Suna Koc, İlhan Akaslan.

**Formal analysis:** Suna Koc.

**Funding acquisition:** Suna Koc.

**Investigation:** Suna Koc.

**Methodology:** Suna Koc, İlhan Akaslan.

**Project administration:** Suna Koc, İlhan Akaslan.

**Resources:** Suna Koc.

**Software:** Suna Koc.

**Supervision:** Suna Koc.

**Validation:** Suna Koc.

**Visualization:** Suna Koc.

**Writing – original draft:** Suna Koc, İlhan Akaslan.

**Writing – review & editing:** Suna Koc.
